# Rapamycin does not alter bone microarchitecture or material properties quality in young-adult and aged female C57BL/6 mice

**DOI:** 10.1093/jbmrpl/ziae001

**Published:** 2024-01-10

**Authors:** Connor C Devine, Kenna C Brown, Kat O Paton, Chelsea M Heveran, Stephen A Martin

**Affiliations:** Chemical and Biological Engineering Department, Montana State University, Bozeman, MT 59718, United States; Mechanical and Industrial Engineering Department, Montana State University, Bozeman, MT 59718, United States; Translational Biomarkers Core Laboratory, Center for American Indian and Rural Health Equity, Montana State University, Bozeman, MT 59718, United States; Biology of Aging Laboratory, Center for American Indian and Rural Health Equity, Montana State University, Bozeman, MT 59718, United States; Mechanical and Industrial Engineering Department, Montana State University, Bozeman, MT 59718, United States; Translational Biomarkers Core Laboratory, Center for American Indian and Rural Health Equity, Montana State University, Bozeman, MT 59718, United States; Biology of Aging Laboratory, Center for American Indian and Rural Health Equity, Montana State University, Bozeman, MT 59718, United States

**Keywords:** animal models, aging, rapamycin, bone formation and resorption, bone biomechanics

## Abstract

Advancing age is the strongest risk factor for osteoporosis and skeletal fragility. Rapamycin is an FDA-approved immunosuppressant that inhibits the mechanistic target of rapamycin (mTOR) complex, extends lifespan, and protects against aging-related diseases in multiple species; however, the impact of rapamycin on skeletal tissue is incompletely understood. We evaluated the effects of a short-term, low-dosage, interval rapamycin treatment on bone microarchitecture and strength in young-adult (3 mo old) and aged female (20 mo old) C57BL/6 mice. Rapamycin (2 mg/kg body mass) was administered via intraperitoneal injection 1×/5 d for a duration of 8 wk; this treatment regimen has been shown to induce geroprotective effects while minimizing the side effects associated with higher rapamycin dosages and/or more frequent or prolonged delivery schedules. Aged femurs exhibited lower cancellous bone mineral density, volume, trabecular connectivity density and number, higher trabecular thickness and spacing, and lower cortical thickness compared to young-adult mice. Rapamycin had no impact on assessed microCT parameters. Flexural testing of the femur revealed that both yield strength and ultimate strength were lower in aged mice compared to young-adult mice. There were no effects of rapamycin on these or other measures of bone biomechanics. Age, but not rapamycin, altered local and global measures of bone turnover. These data demonstrate that short-term, low-dosage interval rapamycin treatment does not negatively or positively impact the skeleton of young-adult and aged mice.

## Introduction

Advancing age is the strongest risk factor for osteoporosis, a skeletal condition characterized by decreased bone mass and quality, which together precipitate skeletal fragility. Osteoporosis affects approximately 60% of adults in early old age (>50 yr),^1^ and its incidence rate is expected to rise as the pace of population aging and life expectancy both increases. The protracted recovery periods of bone fractures in aged adults increase mortality risk and burden the health care system making senile skeletal fragility an important public health issue. Bone is a dynamic tissue that undergoes continual remodeling through the activity of bone-forming osteoblasts and bone-resorbing osteoclasts, which are tightly regulated by a variety of endocrine and paracrine mechanisms such that there is an equal balance of resorption and formation, and total bone mass is maintained under healthy conditions. Aging disrupts skeletal homeostasis by shifting the remodeling balance toward resorption, resulting in decreased bone mass and increased fragility.^2^ Over the past 20 yr, there has been strong push by the geroscience research community to identify interventions that modulate the aging process and decrease vulnerability to aging-related diseases. Rapamycin has emerged as a potential candidate for extending lifespan.^3^ However, it is unclear whether rapamycin has beneficial or detrimental effects to bone quality, architecture, and whole-bone biomechanics and whether the skeletal effects of this intervention change with aging.

In 2009, the FDA-approved macrolide compound rapamycin was identified as the first pharmacological agent to extend maximal lifespan in both sexes in a mammalian species (mice).^4–6^ Rapamycin is an inhibitor of the mechanistic target of rapamycin (mTOR), a serine/threonine kinase complex that regulates cellular growth, proliferation, survival, and energy metabolism; the mTOR pathway has been implicated, through genetic studies, in lifespan control for a variety of experimental organisms including yeast, worms, flies, and mice.^7–9^ Since the initial life-extension findings in 2009, rapamycin has been demonstrated, in rodents, to attenuate age-related diseases and phenotypes including cancer, neurodegeneration, and immune senescence.^10^ The number of preclinical studies investigating rapamycin as a “geroprotective agent” has grown significantly, and several clinical trials are currently underway to determine the safety and impact of rapamycin on indices of aging in humans.^11–13^

Identifying the impact of rapamycin on bone health across the lifespan, as well as the mechanisms through which rapamycin influences bone cell activity, is an important step toward the potential prophylactic use of rapamycin in humans. In vivo and in vitro genetic studies support a role of mTOR signaling in a range of skeletal processes including skeletal development,^14–16^ stem cell dynamics,^17–20^ osteoblast and osteoclast activity,^21–26^ and fracture repair.^27–30^ The results of studies examining the in vivo impact of rapamycin treatment on skeletal health have been equivocal. We previously demonstrated rapamycin treatment impaired bone accrual in young female mice,^31^ and our results are supported by similar findings of impaired skeletal growth in other studies utilizing young animals.^32^^,^^33^ In contrast, in rodent models of senile, ovariectomy-induced, and high turnover–induced osteoporosis, rapamycin, and rapamycin analogs suppressed trabecular bone loss.^34–36^ The variability of results from these studies is likely driven by numerous factors including experimental animal models, rapamycin dosages, dosing schedules, treatment duration, and the skeletal phase (eg, growth, maintenance, senescence) at which the rapamycin treatment was initiated.

Recent work has focused on how specific rapamycin dosing regimens differentially influence systemic metabolic health, which could in-turn influence skeletal health. mTOR exists in 2 distinct complexes: mTOR complex 1 (mTORC1) and mTOR complex 2 (mTORC2). mTORC1 is sensitive to acute rapamycin treatment, and its inhibition drives the longevity and delayed-aging effects of rapamycin treatment. Under chronic rapamycin treatment, however, mTORC2 is also inhibited. This produces deleterious side effects including glucose intolerance and immunosuppression,^37^^,^^38^ which are known to impair skeletal health.^39^ As such, a central goal of rapamycin research is identifying optimal dosing strategies that inhibit mTORC1 but leave mTORC2 signaling intact. In this study, we sought to determine the impact of low-dose (2 mg/kg) interval (1× per 5 d) rapamycin treatment on bone microarchitecture and bone material properties in young-adult and aged female C57BL/6 mice; this dosing regimen has been previously demonstrated to inhibit mTORC1 activity while leaving mTORC2 signaling intact^37^^,^^40^ across a range of tissues, and we hypothesized this regimen would mitigate the detrimental effects of rapamycin on skeletal health we observed in our previous work.

## Materials and methods

### Experimental design

Female C57BL/6 mice were obtained from Jackson Laboratories (3-mo-old mice; *n* = 20) and the National Institute on Aging Aged Rodent Colony (20-mo-old mice; *n* = 18). We chose female mice as our experimental model because (1) postmenopausal females are at the greatest risk of skeletal impairment, and (2) rapamycin treatment for longevity and delayed aging has been demonstrated to have greater efficacy in female rodents.^38^^,^^41^ Mice were housed under controlled conditions. Mice were group housed on a standard light cycle (12-h light; 12-h dark), food and water were provided ad libitum, and body weight was monitored weekly for the duration of the study. All animal procedures were approved by the Montana State University Institutional Animal Care and Use Committee.

Mice were acclimated to the facility for 1 wk prior to study initiation. Following acclimation, 3-mo-old and 20-mo-old mice were randomized into 1 of 2 treatment conditions, rapamycin or vehicle. Mice treated with rapamycin received intraperitoneal injection of rapamycin (dissolved in ethanol then diluted with vehicle containing 5% Tween 80 and 5% PEG4000) at a dosage of 2 mg/kg.^42^ The dosing was given once every 5 d for a duration of 8 wk. Vehicle-treated mice received intraperitoneal injections of equivolume vehicle (saline with 5% Tween 80 and 5% PEG4000) in the same dosing interval and duration.

Mice were approximately 5 mo old and 22 mo old at the conclusion of the treatment regimen when they were deeply anesthetized with 2% isoflurane and bled by cardiac puncture for tissue collection. The right and left femurs were isolated, cleaned of soft tissues, wrapped in saline-saturated gauze, and stored at −20 °C for microcomputed tomography and biomechanical analyses. Left femur length was measured in a consistent orientation using calipers. Left and right tibiae were removed, snap frozen in liquid nitrogen, and stored at −80 °C for molecular analyses.

### Intraperitoneal glucose tolerance test

Intraperitoneal glucose tolerance test (ipGTT) was performed 7 d prior to sacrifice and tissue collection. Mice were fasted for 6 h at the end of their dark cycle. Baseline blood glucose was measured using a glucometer (Accu-check Performa, Roche) with blood collected via tail nick. Following baseline blood glucose measurement, mice were intraperitoneally injected with glucose (2 g/kg). Blood glucose was assessed at 15-, 30-, 60-, and 120-min post glucose injection.

### Microcomputed tomography

Microarchitecture of the left femur was assessed by high-resolution microtomography (μCT40, Scanco Medical AG). μCT image acquisition and analyses adhered to JBMR guidelines.^43^ Scanning parameters included an isotropic voxel size of 10 μm^3^, peak X-ray tube potential of 70 kV, 114 μA tube current, 200 ms integration time, Gaussian filtration, and segmentation. Trabecular microarchitecture in the femoral distal metaphysis was determined from a region 200 μm superior to the proximal side of the distal growth plate that extended 1500 μm proximally with manually contoured endocortical edges. Trabecular bone was identified and selected using manual contouring with a 310 mgHA/cm^3^ threshold. Scanco Trabecular Bone Morphometry evaluation script was used to assess trabecular architecture parameters: trabecular bone volume fraction (Tb.BV/TV, %), trabecular bone mineral density (Tb.BMD, mgHA/cm^3^), specific bone surface (BS/BV, mm^2^/mm^3^), trabecular thickness (Tb.Th, mm), trabecular number (Tb.N, mm^−1^), trabecular separation (Tb.Sp, mm), connective density (Conn.D, 1/mm^3^), and structure model index. Middiaphysis cortical microarchitecture was assessed in 50 equally spaced transverse μCT slices along a 500-μm long region that included the entire outer edge of the cortex. Cortical bone was identified and selected using manual contouring with a 700 mgHA/cm^3^ threshold to compute: total cross-sectional area (bone + medullary area) (Tt.Ar, mm^2^), cortical bone area (Ct.Ar, mm^2^), medullary area (Ma.Ar, mm^2^), bone area fraction (Ct.Ar/Tt.Ar, %), cortical tissue mineral density (Ct.TMD, mgHA/cm^3^), cortical thickness (Ct.Th, mm), cortical porosity (%), and maximum, minimum, and polar moments of inertia (I_max_, I_min_, and J, mm^4^).

### Three-point bending

Right femurs stored at −20 °C were thawed and hydrated with PBS, then placed in a consistent orientation (from operator view: posterior side facing down, distal facing left) on a custom fixture with 2 square-notch contact points with an 8-mm span. A load was applied at 5 mm/min until failure (Instron 5543). Hydration was maintained before and during testing using PBS. Sample data were excluded if the position of the bone observably deviated in the loading span during testing. Stress vs strain plots were generated from load and displacement data using geometric measures from μCT (Imin and Cmin) and the standard equations for testing bone flexural properties^44^ in a custom MATLAB code. Outcomes from the load–displacement data included stiffness, ultimate load, fracture load, and energy at fracture (area under load–displacement curve until fracture). Yield was defined at the intersection of a secant line drawn with a 10% reduction in stiffness and the load–displacement curve. Apparent material properties, including elastic modulus, yield stress, postyield strain, and toughness, were estimated using standard beam bending equations.^44^ Peak bending moment and section modulus were calculated to discern the contributions to bone strength from geometry versus apparent material properties.

### Immunoblotting and protein multiplex

Tibiae were pulverized in liquid nitrogen, and total cellular protein was extracted using an Invitrogen PARIS isolation kit (AM1921) according to the manufacturer’s protocol. Proteins were detected by immunoblotting using standard techniques. Forty micrograms of protein were separated by SDS-PAGE electrophoresis using Bio-Rad Mini-PROTEAN TGX AnyKd Gels (4 569 036). Separated proteins were transferred to PVDF membranes using a Bio-Rad Transblot-Turbo System. Membranes were blocked and incubated at 4 °C overnight with respective primary antibodies. Following primary antibody incubation, membranes were washed and incubated with HRP-conjugated secondary antibodies for 1 h at room temperature. Membranes were then washed and developed with Bio-Rad Clarity Western ECL Substrate (170–5060) and imaged using a Protein Simple Fluoro-ChemR Imaging System. Quantification was performed by densitometry using Adobe Photoshop software. Antibodies used were S6 Ribosomal Protein (2217; Cell Signaling Technology; 1:1000 dilution in Tris-Buffered Saline with 0.1% Tween 20 [TBST] and 5% bovine serum albumin [BSA]), serine 240/244 phospho-S6 Ribosomal protein (2215; Cell Signaling Technology; 1:1000 dilution in TBST with 5% BSA), AKT (4691; Cell Signaling Technology; 1:1000 dilution in TBST with 5% BSA), serine 473 phospho-AKT (4060; Cell Signaling Technology; 1:2000 dilution in TBST with 5% BSA), Osteoprotegerin (OPG; AF459; R&D Systems; 1:1000 dilution in TBST with 5% nonfat milk), and RANKL (sc-377 079; Santa Cruz Biotechnology; 1:200 dilution in TBST with 5% BSA).

Protein multiplex analysis was conducted using a magnetic bead-based Luminex Mouse Discovery Assay per the manufacture’s protocol (LXSAMSM-10; R&D Systems). The assessed proteins were TNFα, IL-1β, IL-6, MCP1, RANTES, Osteopontin, MMP2, MMP3, MMP8, and MMP12. About 30 μg of protein was loaded per sample, and the analysis was performed using a Bio-Rad BioPlex 200 instrument.

### Serum biochemistry

Serum P1NP was measured using a Mouse P1NP ELISA kit (MBS703389; MyBiosource), and serum CTX-1 was measured using Mouse CTX-1 ELISA kit (MBS722404; MyBiosource) according to the respective manufacturer’s protocol.

### Statistics

Two-factor ANOVA tested the effects of treatment (rapamycin or vehicle control), age (5 mo and 22 mo), and their interaction on measures of bone structure, biomechanics, protein expression, and serum analyses. Residual analysis, Levene’s test for homogeneity of variance, and Anderson–Darling tests of normality were used to assess goodness of model fit. Differences were considered significant at *P* ≤ .05. Outliers were identified using the fence method (Q3 + 1.5IQR) and verified using 2-tailed Grubbs for minimum and maximum outliers. Removal of outliers did not impact the interpretation of the data except for tibia IL-6 protein expression (Figure 3G), and all figures are presented with outliers included unless otherwise stated. All data are presented as mean ± SEM. Data analysis was performed using Minitab version 21.3.1 and R version 3.4.3.

## Results

### Body mass and ipGTT

**Figure 1:**
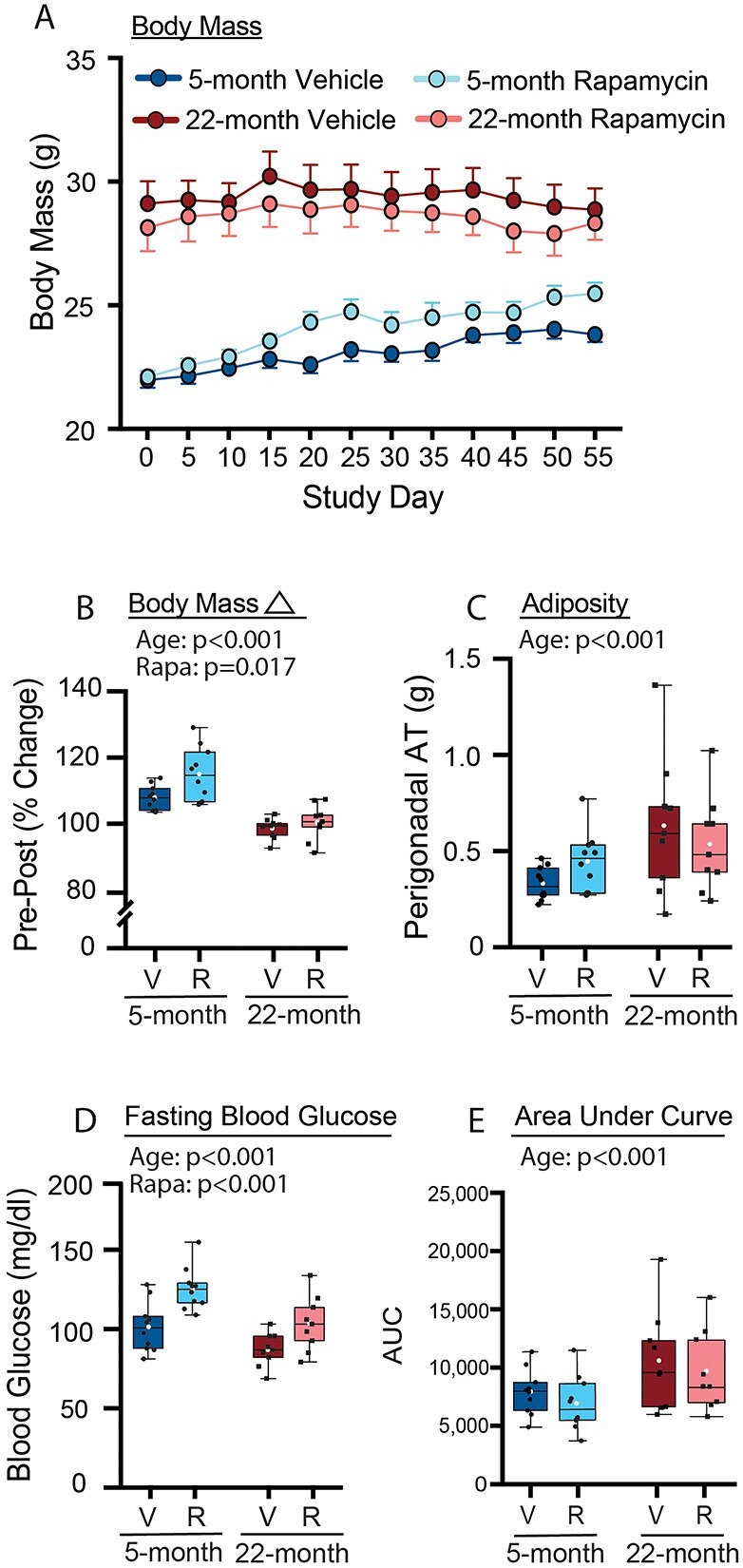
Effects of age and rapamycin on (A) body mass, (B) body mass change, (C) adiposity, (D) fasting blood glucose, and (E) ipGTT area under the curve in 5- and 22-mo-old female C57BL/6 mice following 8 wk of treatment with rapamycin (2 mg/kg; 1 × 5 d) or vehicle. Data are presented as mean (white circle), median (line), 25^%^/75^%^ IQR (bottom and top of box), and minimum/maximum range (whiskers), with individual values (black circles) overlayed.

Aged female mice had higher baseline and terminal body mass compared to young-adult mice (Figure 1A). Mice treated with rapamycin gained significantly greater body mass compared to vehicle-treated mice over the course of the study, and young-adult mice gained greater body mass compared to aged mice (Figure 1B). Perigonadal adipose tissue mass was higher in aged mice compared to young-adult mice (Figure 1C), but adiposity was unchanged by rapamycin treatment, suggesting that the rapamycin-induced increase in body mass was likely due to increased lean mass. Chronic rapamycin treatment has been previously shown to induce insulin resistance and glucose intolerance in mice,^37^^,^^40^ which are known effectors of skeletal impairment. We conducted an ipGTT 1 wk prior to euthanasia to determine the impact of aging and rapamycin on glucose tolerance. Aged mice had a lower fasting blood glucose compared to young-adult mice, and rapamycin-treated mice had a higher fasting glucose compared to vehicle-treated mice (Figure 1D). Analysis of the blood glucose area under the curve (AUC) revealed no differences between ages or treatment (Figure S1 A&B). However, when the AUC was corrected for baseline blood glucose, aged mice had a significantly higher glucose AUC compared to young-adult mice (Figures 1E and S1C). There were no interactions for blood glucose variables between mouse age and treatment.

### Bone morphology and microarchitecture

There was no impact of age on femur length measured by calipers, indicating the young-adult mice in this study were skeletally mature at the time of tissue harvest, and rapamycin did not influence bone accrual. Bone microarchitecture is an indicator of overall bone health and one determinate of bone strength. We utilized μCT to assess cortical geometry and trabecular bone microarchitecture in the femur (Table 1 and Figure S2 B&C). The initiation of rapamycin treatment occurred at 3 mo of age in our young-adult mouse group, which is a time-point after skeletal maturity is achieved.^45^ As expected, aging had a significant effect on both cortical bone morphology and trabecular bone microarchitecture.^46^ Compared with young-adult mice, aged mice had higher cortical cross-sectional area and decreased cortical thickness. Aging also reduced cancellous bone volume fraction, bone mineral density, connectivity density, trabecular number, and increased trabecular spacing and thickness in the distal femur metaphysis compared to young-adult mice. The age-related reduction in cancellous bone and increased trabecular spacing was primarily due to decreased trabecular number, as the trabecular thickness was higher in aged mice compared to young-adult mice. A potential explanation for the higher trabecular thickness in aged mice is at 22 mo of age, their thinner trabecular have already been resorbed, and only the thicker trabeculae remain; whereas in the young-adult mice, there are nearly 2-fold higher number of trabeculae, which are thinner, but cumulatively contain a much greater surface area. There were no independent effects of rapamycin or age × treatment interactions on femur cortical geometry or bone microarchitecture.

**Table 1:** Effects of age and rapamycin on trabecular microarchitecture and cortical geometry in 5- and 22-mo-old female C57BL/6 mice following 8 wk of treatment with rapamycin (2 mg/kg; 1 × 5d) or vehicle. Data are presented as mean and SD.

**Variable**	**5-Month-Old**		**22-Month-Old**		**Age**	**Rapamycin**	**Age × Treatment**
	**Vehicle** **(n = 10)**	**Rapamycin** **(n = 10)**	**Vehicle** **(n = 9)**	**Rapamycin** **(n = 9)**	***P* value**	***P* value**	***P* value**
***Total Femur***							
Length (mm)	15.89 ± 0.29	15.75 ± 0.22	15.7 ± 0.21	15.75 ± 0.16	NS	NS	NS
***Femur metaphysis (cancellous bone)***							
BV/TV (%)	5.74 ± 2.51	6.67 ± 1.38	2.61 ± 4.07	2.8 ± 3.09	(p < 0.001)	NS	NS
BMD (mgHA/cm^3^)	112.58 ± 20.07	118.31 ± 13.94	76.02 ± 33.51	72.58 ± 19.85	(p < 0.001)	NS	NS
BS/BV (mm^2^/mm^3^)	58.74 ± 7.52	55.08 ± 5.48	49.6 ± 7.64	49.41 ± 3.89	(p < 0.001)	NS	NS
Conn.D (1/mm^3^)	31.94 ± 23.09	32.16 ± 14.14	13.67 ± 36.63	12.08 ± 20.32	(p = 0.022)	NS	NS
SMI	3.28 ± 0.37	3.28 ± 0.23	3.37 ± 0.64	3.08 ± 0.84	NS	NS	NS
Tb.N (mm^-1^)	3.01 ± 0.55	3.25 ± 0.42	1.54 ± 0.63	1.68 ± 0.39	(p < 0.001)	NS	NS
Tb.Th (mm)	50 ± 4	53 ± 5	58 ± 10	54 ± 3	(p = 0.041	NS	NS
Tb.Sp (mm)	345 ± 79	314 ± 47	730 ± 229	627 ± 111	(p < 0.001)	NS	NS
***Femur diaphysis (cortical bone)***							
Ct.Ar (mm^2^)	0.81 ± 0.06	0.81 ± 0.03	0.8 ± 0.04	0.8 ± 0.09	NS	NS	NS
Ma.Ar (mm^2^)	0.85 ± 0.11	0.79 ± 0.04	1.32 ± 0.21	1.39 ± 0.1	(p < 0.001)	NS	NS
Tt.Ar (mm^2^)	1.66 ± 0.15	1.6 ± 0.05	2.12 ± 0.22	2.19 ± 0.16	(p < 0.001)	NS	NS
Ct.Ar/Tt.Ar	48.75 ± 2.01	50.65 ± 1.22	38.06 ± 4.7	36.32 ± 2.55	(p < 0.001)	NS	NS
Ct.Th (mm)	195 ± 7	200 ± 5	167 ± 12	163 ± 16	(p < 0.001)	NS	NS
Ct.TMD (mgHA/cm^3^)	1259.17 ± 22.72	1262.32 ± 13.47	1251.37 ± 12.21	1246.73 ± 22.79	NS	NS	NS
Ct.Porosity (%)	0.78 ± 0.09	0.78 ± 0.07	0.83 ± 0.09	0.79 ± 0.09	NS	NS	NS
pMOI (mm^4^)	0.34 ± 0.06	0.33 ± 0.02	0.45 ± 0.06	0.47 ± 0.09	(p < 0.001)	NS	NS
Imax (mm^4^)	0.23 ± 0.04	0.22 ± 0.02	0.29 ± 0.03	0.29 ± 0.06	(p < 0.001)	NS	NS
Imin (mm^4^)	0.11 ± 0.02	0.1 ± 0.01	0.17 ± 0.03	0.18 ± 0.03	(p < 0.001)	NS	NS

### Three-point bending

**Figure 2:**
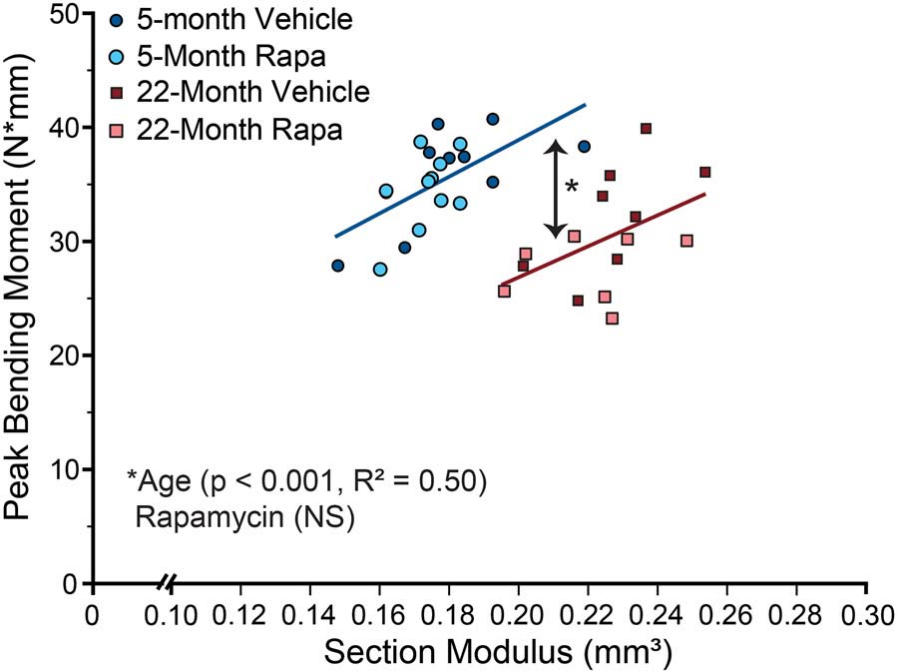
Effects of age and rapamycin on the linear relationship between section modulus and peak bending moment in 5- and 22-mo-old female C57BL/6 mice following 8 wk of treatment with rapamycin (2 mg/kg; 1 × 5 d) or vehicle.

Whole-bone flexural strength is the result of hierarchical contributions to fracture resistance including structure, mineral content, and matrix quality.^47^ We used 3-point bending to assess whole-bone strength and energy dissipation, as well as to estimate bone material properties (Table 2). Compared with young adult mice, aged mice had significantly lower whole-bone strength (ultimate stress, peak bending moment), yield strength, elastic modulus, and higher postyield displacement. There was a trend for lower ultimate stress in rapamycin-treated mice for both ages (rapamycin main effect, *P* = .064), but it did not reach statistical significance. Changes to cortical bone geometry did not fully explain observed age-related differences in whole-bone strength, indicating bone material strength was altered with age (Figure 2). The linear relationship between section modulus and peak bending moment for aged mice had a lower intercept than young-adult mice, indicating a 32.3% reduction in aged mice whole-bone strength compared to young-adult mice. Rapamycin did not affect whole-bone flexural properties or the linear relationship between peak bending moment and section modulus at either age. Of note, a 22-mo-old vehicle-treated mouse was excluded from this analysis for having a low outlier section modulus, and a 22-mo-old rapamycin-treated mouse was also excluded for having a high outlier peak bending moment. Statistical interpretation of these data did not change when outliers were removed and presented *P*-values are for the data excluding outliers.

**Figure 3:**
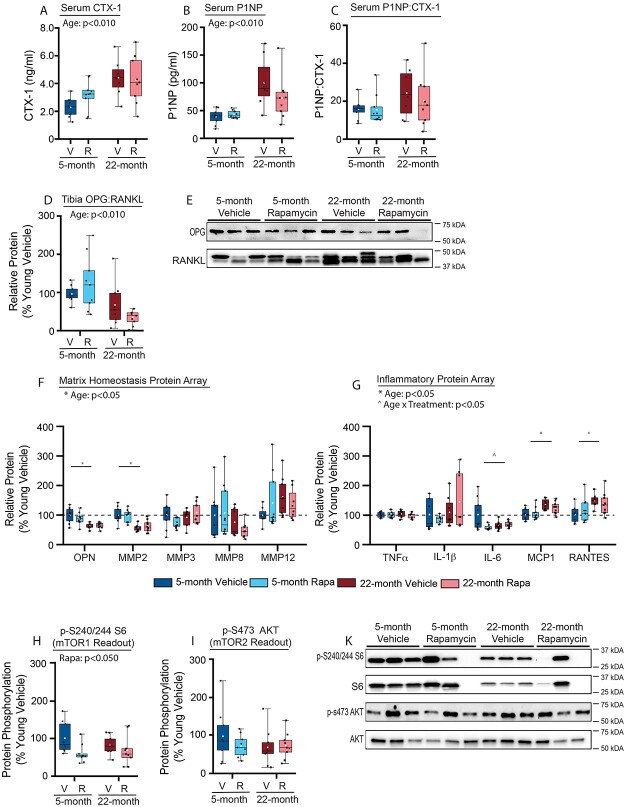
Effects of age and rapamycin on (A) serum CTX-1, (B) serum P1NP, (C) serum P1NP:CTX-1, (D and E) immunoblotting analysis and quantification of tibia OPG:RANKL, (F) “matrix-homeostasis” multi-plex protein array, (G) “inflammatory” multi-plex protein array, (H-K) immunoblotting analysis, and quantification of phosphorylated S6 (S240/244) and AKT (S473) in tibia of 5- and 22 mo-old female C57BL/6 mice following 8 wk of treatment with rapamycin (2 mg/kg; 1 × 5d) or vehicle. Data are presented as mean (white circle), median (line), 25^%^/75^%^ IQR (bottom and top of box), and minimum/maximum range (whiskers), with individual values (black circles) overlayed.

**Table 2:** Effects of age and rapamycin on femur whole-bone mechanical properties and estimated tissue material properties in 5- and 22-mo-old female C57BL/6 mice following 8 wk of treatment with rapamycin (2 mg/kg; 1 × 5d) or vehicle. Data are presented as mean and SD.

**Variable**	**5 Mo old**	**Rapamycin (*n* = 10)**	**22 Mo old**		**Age**	**Rapamycin**	**Age × treatment**
	**Vehicle (*n* = 10)**		**Vehicle (*n* = 9)**	**Rapamycin (*n* = 9)**	***P* value**	***P* value**	***P* value**
**Whole-Bone Mechanical Properties**							
Stiffness (N/mm)	90.52 ± 14.17	93.24 ± 7.49	97.64 ± 13.47	86.16 ± 8.11	NS	NS	NS
Peak bending moment (N*mm)	35.85 ± 4.29	34.46 ± 3.39	33.53 ± 5.87	29.29 ± 5.39	.023	NS	NS
Ultimate load (N)	17.92 ± 2.14	17.23 ± 1.7	16.77 ± 2.94	14.64 ± 2.7	.023	NS	NS
Yield load (N)	8.3 ± 2.28	7.26 ± 0.62	6.51 ± 0.54	7.16 ± 0.95	.040	NS	NS
Post-yield displacement (mm)	305.7 ± 194.6	372.9 ± 150.3	644.9 ± 293.3	534.6 ± 316.5	.004	NS	NS
Work to fracture (mJ)	4.85 ± 2.1	5.17 ± 2.13	6.95 ± 3.66	4.43 ± 2.94	NS	NS	NS
**Estimated Tissue Material Properties**							
Modulus (GPa)	8.49 ± 1.68	8.62 ± 2.03	7.18 ± 2.54	5.94 ± 2.1	.007	NS	NS
Ultimate strength (MPa)	200.04 ± 18.97	190.4 ± 35.24	162.27 ± 48.31	133.02 ± 18.14	.001	NS	NS
Yield strength (MPa)	94.01 ± 30.94	79.67 ± 10.86	62.85 ± 13.98	66.21 ± 13.88	.002	NS	NS
Toughness (N/mm^2^)	6.49 ± 2.64	6.82 ± 2.79	8.9 ± 4.99	5.61 ± 3.79	NS	NS	NS

### Serum and tibia proteins

Aged-related skeletal impairment is due, in part, to unbalanced bone resorption and bone formation. The cause of dysregulated skeletal remodeling during aging is multifaceted and includes altered osteoblast/osteoclast/osteocyte survival and activity, stem cell differentiation dynamics, and alterations in matrix properties.^48^ We assessed serum CTX-1 and P1NP, biomarkers of bone resorption and bone formation, respectively, to identify the impacts of age and rapamycin on global bone turnover (Figure 3A-C). Aged mice had higher levels of both serum CTX-1 and P1NP compared to young-adult mice, but the P1NP:CTX-1 ratio was not different between ages. There was no impact of rapamycin on serum CTX-1, P1NP, or P1NP:CTX-1, nor were there age × treatment interactions. At the local skeletal tissue level, the ratio of OPG and RANKL reflects the balance of bone formation (OPG) and resorption (RANKL). OPG is secreted by osteoblasts and osteocytes and acts as a decoy receptor for RANKL, which inhibits its interaction with the osteoclast RANK receptor and prevents activation of bone resorption. Young mice exhibited a significantly higher OPG:RANKL ratio compared to aged mice, indicating the bone turnover balance in the tibia likely favored bone formation in the young-adult mice compared to aged mice (Figure 3D and E). This ratio was not impacted by rapamycin, and there was no age × treatment interaction. We also assessed the expression of proteins involved in the maintenance of matrix homeostasis (Figure 3F), which is known to be dysregulated during aging and associated impaired bone quality. The expression of OPN and MMP2 was lower in aged mice compared to young-adult mice. OPN is an important regulator of biomineralization, and MMP2 is central to collagen degradation; reduced expression of these enzymes in 22-mo-old mice likely indicates diminished frequency of remodeling events, which is consistent with the accumulation of mineral content in skeletal tissue during aging.^48^ Rapamycin did not impact the expression levels of these specific proteins, and there were no age × treatment interactions.

Age-related inflammation is believed to be mechanistically involved in senile skeletal impairment,^49^ and rapamycin treatment has been demonstrated to attenuate age-associated inflammation across a range of tissues.^50^ Assessment of prototypical inflammatory mediators revealed the expression of proinflammatory factors MCP1 and RANTES was higher in aged mice compared to young-adult mice; additionally, there was an age × treatment interaction for IL-6 where rapamycin-treated young-adult mice had lower IL-6 expression compared to vehicle-treated young-adult mice, but this effect was not present in aged mice (Figure 3G). Of note, the age × treatment interaction for IL-6 was only significant when 4 outliers (YR, YR, AR, AV), identified by the fence method, were excluded. There was no impact of age, rapamycin, or interaction on TNFα or IL-1β expression. Together, these data confirm aging is associated with a low-grade inflammatory response in the tibia, which was not impacted by rapamycin treatment. Lastly, we assessed mTOR signaling in the tibia to determine the impact of our rapamycin treatment on inhibition of mTORC1 and mTORC2 (Figure 3H-K). Both young and aged mice treated with rapamycin exhibited reduced phosphorylation of serine 240/244 on S6, a downstream target of mTORC1’s kinase activity, while there was no impact of rapamycin on the phosphorylation of serine 473 on AKT, a downstream target of mTORC2’s kinase activity. These findings indicate that in the tibia, our short-term, low-dose, interval rapamycin treatment regimen inhibited mTORC1 while leaving mTORC2 signaling intact.

## Discussion

Here, we report that deleterious changes to bone microarchitecture and femur material properties that occur with aging in female C57BL/6 mice are neither exacerbated nor ameliorated by 8 wk of low-dosage, interval rapamycin treatment. The observed age-related changes to bone microarchitecture, geometry, flexural strength, and bone metabolism in female C57BL/6 mice are consistent with previous literature^46^; nonetheless, these findings provide additional data regarding skeletal biology in aging female mice, which are understudied compared to male mice.

As advancing age is the top risk factor for fracture risk and fracture-related comorbidities, understanding how geroprotective agents, such as rapamycin, influence the skeletal physiology across the lifespan is critical for potential prophylactic usage as antiaging therapies. The dosage, dosing schedule, delivery route, and the age or life time-point to initiate rapamycin treatment are under intense study.^37^^,^^40^^,^^51^ The first evidence of rapamycin’s geroprotective effects in rodents came in a 2009 report from the National Institute on Aging (NIA) Interventions Testing Program (ITP), where they demonstrated rapamycin encapsulated in food (14 ppm; ~2 mg/kg) extended lifespan in mice in a sex-specific manner, with female mice exhibiting a greater benefit compared to male mice. However, this regimen produced numerous undesirable side effects including cataracts, glucose intolerance, insulin resistance, gastrointestinal disorders, and immunological consequences. It has since become evident that chronic treatment with rapamycin inhibits both mTORC1 and mTORC2, and multiple studies have demonstrated that adverse effects are largely due to inhibition of mTORC2.^40^ As such, researchers have been exploring whether reduced dosages and intermittent/transient scheduling can inhibit mTORC1 but not mTORC2, to produce geroprotective effects while limiting adverse effects.^37^^,^^40^

We previously reported rapamycin treatment at a dosage of 4 mg/kg, given via intraperitoneal injection every other day, for a duration of 12 wk, was detrimental to the skeleton of 8-wk-old female mice.^31^ We speculated that these effects were due to the young age at which rapamycin was initiated and/or the dosing regimen inducing potential mTORC2 inhibition in bone cells. mTORC2 inhibition could have impacted the skeleton either directly via alterations in bone cell dynamics regulated by mTORC2 or indirectly via systemic perturbations such as impaired glucoregulation. Our work here builds upon that study by demonstrating rapamycin treatment at a lower dosage, shorter duration, and greater interval, which induced minimal metabolic dysfunction, did not have deleterious effects to the skeleton of young-adult or aged female C57BL/6 mice. Furthermore, we show that our treatment regimen was successful in significantly reducing mTORC1 signaling, while leaving mTORC2 signaling intact in the tibia, suggesting that the observed mTOR1 inhibition was either insufficient to induce skeletal changes, and/or mTOR1 inhibition is uncoupled from bone microarchitecture and material properties in the femur, as we observed no significant rapamycin effects in our μCT and 3-point bending analyses. These findings are important because (1) they demonstrate an absence of negative consequences on the skeleton using a rapamycin regimen that has been shown to have antiaging effects^40^ and (2) define the lower end of rapamycin dosing from which future work can build upon to optimize skeletal health and delay aging, simultaneously.

Review of the literature regarding in vivo rapamycin studies indicates that both the treatment regimen and animal model are important variables that likely mediate the skeletal response to rapamycin. In young, wild-type animals that are growing or have just reached skeletal maturity, rapamycin tends to retard bone growth and accrual, induce bone loss, and dysregulate growth plate dynamics, likely through inhibition/antagonism of anabolic signaling pathways (eg, insulin, IGF-1, growth hormone).^31–33^ Alternatively, only 2 studies have examined the impact of rapamycin on the skeleton of aged animals and, in contrast to our work presented here, both studies have demonstrated geroprotective effects of rapamycin. Luo et al. determined 12 wk of rapamycin (intraperitoneal injection; 1 mg/kg; daily) in 24-mo-old male Sprague Dawley rats protected against age-related cancellous bone loss in the tibia and vertebrate.^35^ We speculate the lack of rapamycin effect on aged bone in our study, compared to Luo et al., could be due to either the rapamycin dosing regimen or the animal model. Although we utilized a higher rapamycin dosage, our study duration was shorter, and we used an interval schedule, which may not have provided a sufficient stimulus to induce effects. Alternatively, species-related differences between mice and rats may have also contributed to the discrepancy in results as the temporal regulation of bone accrual and loss throughout life varies between mice and rats. In a second study, An et al. showed 9 wk of dietary rapamycin (42 ppm; ~7 mg/kg/d) in female C57BL/6 mice reversed age-associated alveolar bone loss and attenuated age-associated periodontal inflammation.^52^ The etiology of age-associated periodontal disease is largely driven by localized inflammation, which is unique from age-related bone loss that occurs in weight-bearing bones; thus, direct comparison between this study and ours may be inappropriate. Lastly, several reports have demonstrated that rapamycin treatment attenuates skeletal dysfunction in rodent models with perturbations that induce severe bone loss (eg, ovariectomy, lipopolysaccharide [LPS] injection, advanced oxidation protein products injection).^34–36^ Although these studies are critical for defining relevant biological mechanisms through which rapamycin impacts skeletal physiology, the direct translation of these models to normal human aging remains to be established.

The present study has several limitations. This study focused on rapamycin treatment at only 2 time-points (3 mo and 20 mo old at beginning of treatment), and it is unclear how this rapamycin treatment regimen may influence skeletal biology if applied at a different life phase (eg, ~12 mo old; middle aged). Mice begin slowly losing cancellous bone at ~6 mo of age, and once they have reached what is broadly considered an aged phenotype, much of their cancellous bone at some clinically relevant skeletal sites (eg, distal femur) has been resorbed.^46^^,^^53^ This is particularly the case with C57BL/6 mice, which have the lowest bone density and volume compared to similar inbred mouse strains.^45^ An efficacious treatment window for this strain of mice might require starting treatment at a much younger age. We utilized a low dosage, interval, 8-wk rapamycin treatment duration with the goal of minimizing systemic metabolic perturbations; however, it is possible that this regimen may not have provided a sufficient stimulus to induce effects. Finally, we performed this study in female mice because (1) postmenopausal females are at the greatest risk of skeletal impairment and (2) rapamycin treatment for longevity and delayed aging has had a greater demonstrated efficacy in female rodents.^38^^,^^41^ The findings from this study may not be applicable to male mice due to well established sex differences in skeletal physiology.^54^^,^^55^

In summary, we demonstrate that 8 wk of rapamycin at a low-dosage treatment regimen does not impact several key features of skeletal aging, including microarchitecture or whole-bone flexural biomechanics. As the human population rapidly ages, identifying the impact of geroprotective agents, such as rapamycin, on skeletal physiology is imperative as bone health during aging influences both quality of life and mortality. Continuing to incrementally characterize the subtleties of rapamycin treatment (ie, dosage, dosage schedule, route of delivery, age of initiation) on skeletal physiology will allow researchers to better appreciate its potential prophylactic usage as an antiaging therapy in humans.

## Supplementary Material

Figure_S1_ziae001

Figure_S2_ziae001

## Data Availability

The data that support the findings of this study are available from the corresponding author upon reasonable request.
