# Supplementary figures and images for: Rapamycin does not alter bone microarchitecture or material properties quality in young-adult and aged female C57BL/6 mice
Source: JBMR Plus. 2024 Jan 10;8(2):ziae001. doi: 10.1093/jbmrpl/ziae001 (PMC10945714; doi:10.1093/jbmrpl/ziae001)

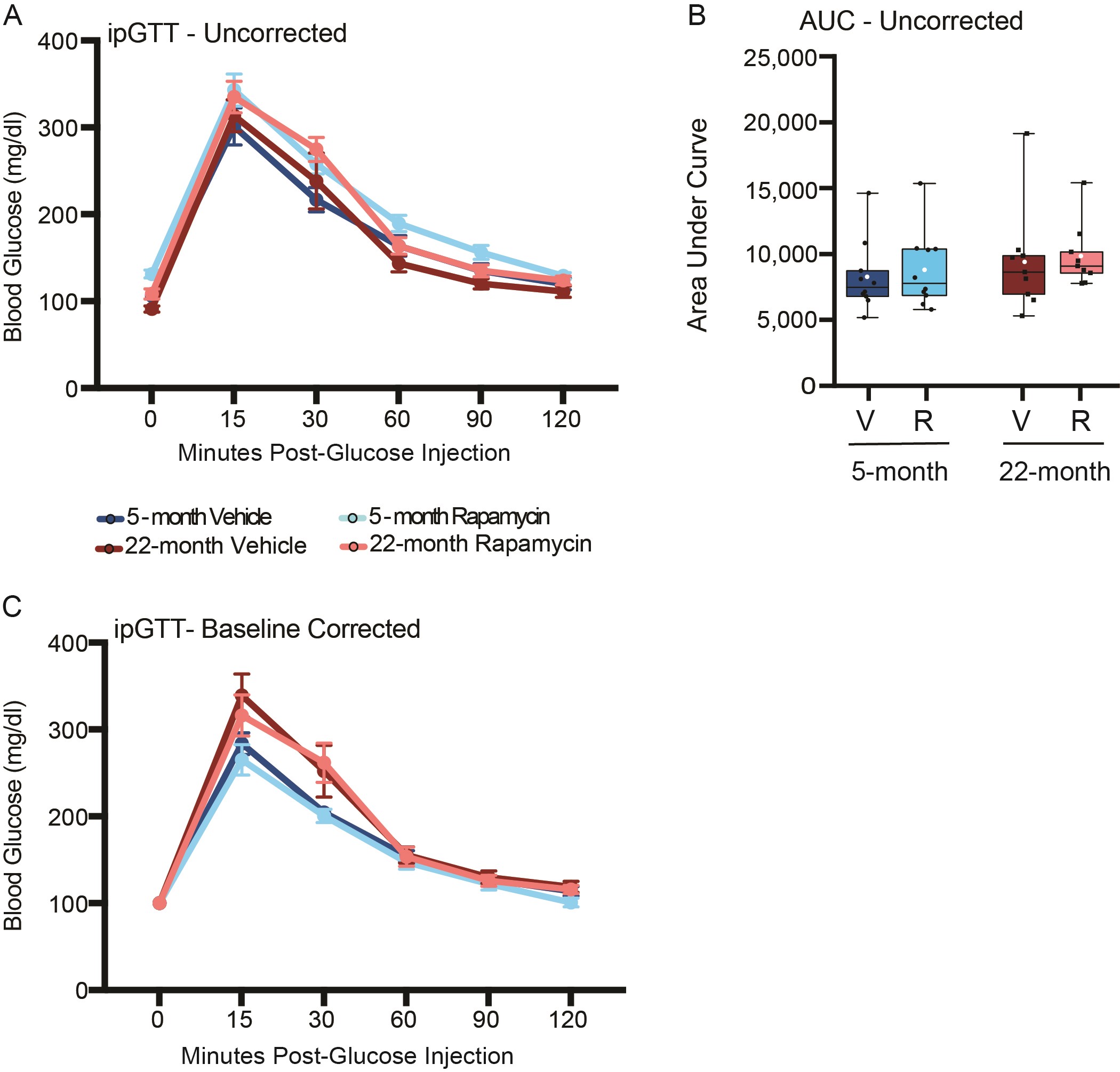

Supplement: Figure_S1_ziae001 [file figure_s1_ziae001.jpeg]

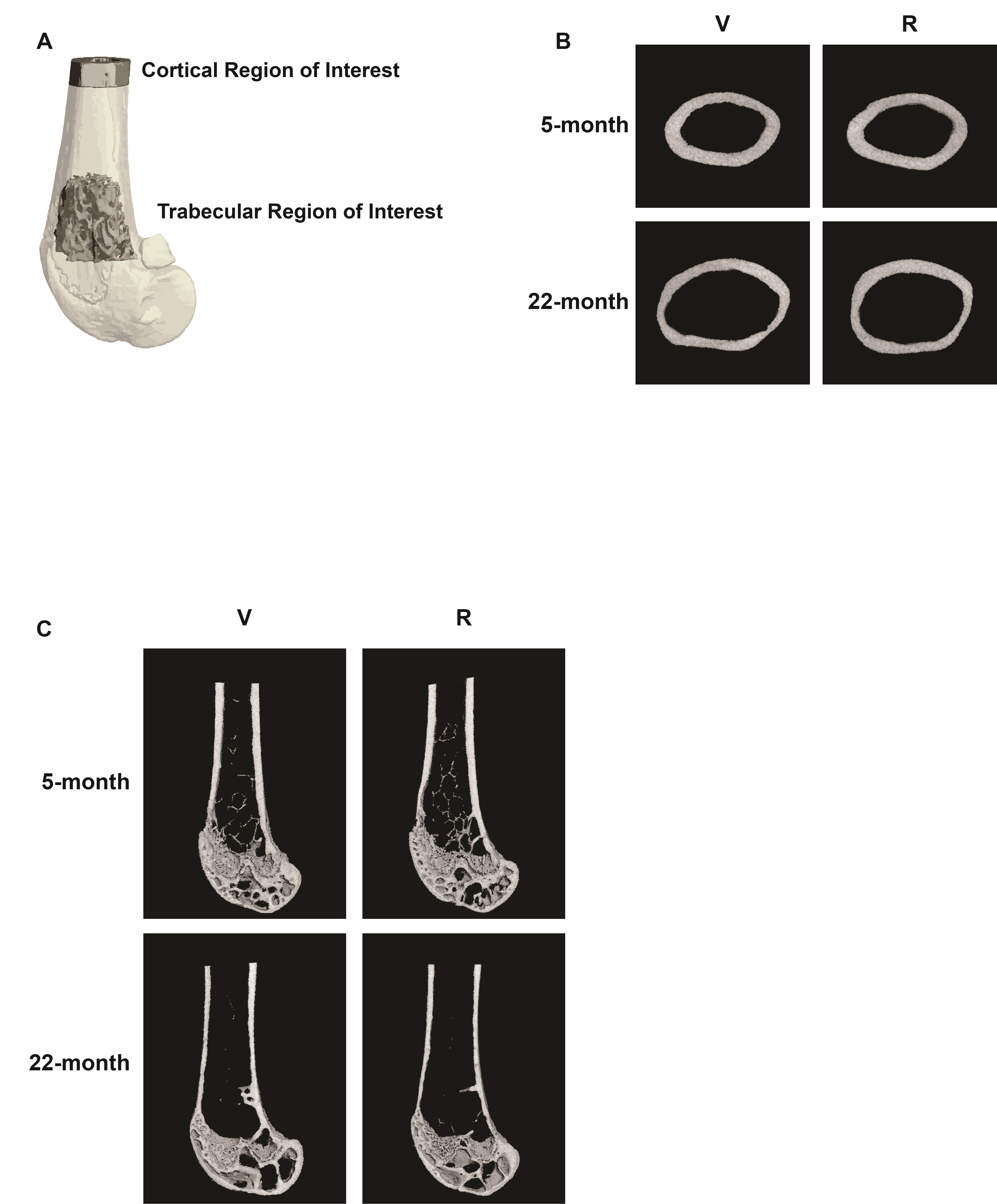

Supplement: Figure_S2_ziae001 [file figure_s2_ziae001.jpeg]
